# Chlorine Gas as a Lewis Acid–Base Probe for Molten Salts of Divalent Metal Ions

**DOI:** 10.1021/acs.jpcb.6c01313

**Published:** 2026-06-12

**Authors:** Yang Chen, Matthew S. Emerson, Hung H. Nguyen, Raphael Ogbodo, Vyacheslav S. Bryantsev, James F. Wishart, Claudio J. Margulis

**Affiliations:** † Department of Chemistry, 4083 The University of Iowa, Iowa City, Iowa 52242, United States; ‡ Chemistry Department, 8099 Brookhaven National Laboratory, Upton, New York 55455, United States; § Chemical Sciences Division, 6146 Oak Ridge National Laboratory, Oak Ridge, Tennessee 37831, United States

## Abstract

In the context of energy applications for salts and, more specifically, in the case of molten salt reactors, the solvation of corrosion species, the nature and behavior of radiation-produced excess electrons, transient radicals, and molecular gas species all depend on the Lewis acid–base behavior of the constituent salt melt. Speciation of dissolved species and their transport properties are also influenced by the ability of the melt to form networks. This article focuses on the structural properties of melts composed of alkaline earth metal ions coupled with the Cl^–^ anion, and the quantum mechanical behavior of Cl_2_, a typical product of the reaction of radiation-produced chlorine radicals in Cl^–^-based molten salts. We explore the effect of M^2+^ Lewis acidity on chlorobasicity, seen in this work as the availability of Cl^–^ ions to chemically react with Cl_2_ to produce Cl_3_^–^.

## Graphical abstract

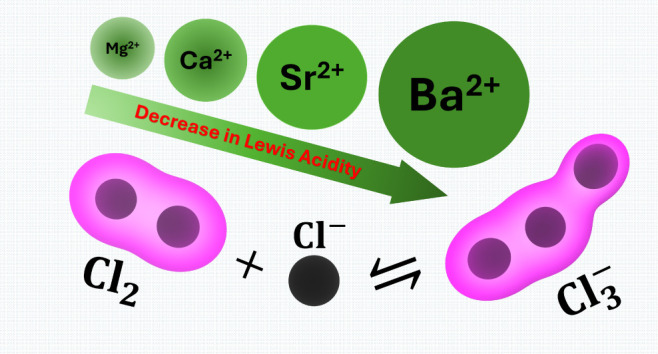

## 1 Introduction

The global energy landscape has undergone a fundamental transformation since the mid-20th century, characterized by a burgeoning demand for high-efficiency power generation. This transition necessitates the development of advanced energy technologies, including, among these, modern Molten Salt Reactors (MSRs), high-temperature electrochemical storage systems, and third-generation concentrated solar power (CSP) stations.^1,2^ At the chemical core of these applications are molten salts, nonmolecular liquids with high thermal stability. Any such system, when exposed to the harsh environments resulting from radiation and high temperature, may present complex chemical challenges, including the appearance of excess electrons^3–11^ and reactive hole species,^8–16^ which, in ionic liquids and molten salts, originate as neutral radicals, as they stem from anions losing an electron. These neutral radicals (in our case, Cl^·^, which converts to Cl_2_^·–^) can react to form halogenic gas that may contribute to corrosivity.^8,10,14^

In these anhydrous ionic environments, the absence of a protic component shifts the chemical focus toward the concept of Lewis acidity and its direct impact on chlorobasicity. The chemical behavior of molten chlorides toward dissolved species is often defined by the ability of Cl^–^ to donate electron density, and this, in turn, is affected by the polarizing nature of the cations. Within this framework, metal ions (particularly multivalent cations) function as primary Lewis acids, modulating the electron-donating power of the surrounding anions.^17^ High Lewis acidity in the cations effectively moderates the basicity of the Cl^–^ ions, sequestering them into “coordination” and electron sharing (or polarization),^18,19^ which often takes the form of Cl^–^-decorated multivalent cationic networks.^20–22^ Put differently, in chloride-based molten salts, Lewis acidity is related to structure and the freedom of Cl^–^ species to donate density to other dissolved species^23^ including those resulting from corrosion and radiation.

Historically, it was noticed in early studies^24^ in the context of gas solubility and the electrolysis of MgCl_2_ to produce magnesium metal that Cl_2_ has a distinctly different spectrum in neat MgCl_2_ when compared to a eutectic mixture of LiCl and KCl, and this was empirically ascribed to the possible chemical equilibrium between Cl_2_ and Cl_3_^–^. In the current study, as a function of cationic Lewis acidity and melt structure,^25–32^ we explore the ability of Cl^–^ to react with Cl_2_ from a first-principles perspective.^14^

## 2 Methods

To gain a thorough understanding of structure in the neat divalent melts, we used the Polarizable Ion Model (PIM), which is the best and most reasonable representation for molecular dynamics (MD) simulation boxes with thousands of ions.^33^ To study chemical reactivity in these melts, we equilibrated smaller PIM simulation boxes and then used these as initial conditions for first-principles trajectories. Details and protocols are described in the following subsections.

### 2.1 Classical Simulations

Molecular dynamics simulations of alkaline-earth chloride molten salts using the PIM were performed using the Metalwalls software package (version 20.05).^34^ All simulations were performed in periodic cubic boxes using the three-dimensional Ewald summation method implemented in Metalwalls. PIM force field parameters for CaCl_2_, SrCl_2_, and BaCl_2_, which include ionic polarizabilities, charges, charge-dipole damping functions, and Born-Mayer-Huggins (BMH) potential terms, were taken from Ishii et al.,^33^ while parameters for MgCl_2_ were taken from Wu et al.^29^ Random initial input positions were generated using the PACKMOL software^35^ to contain 1000 alkaline-earth cations and 2000 chloride anions. The real and reciprocal space Ewald summation tolerances were set to 1.63e-5 and 1.0e-7, respectively, with a real-space cutoff of 22.677 Bohr (12 Å). Induced dipoles were evaluated self-consistently at each time step by conjugate gradient minimization using a convergence threshold of 1.0e-7. PIM simulations in this study used an identical equilibration procedure described previously.^21^ Briefly, a short nonpolarizable simulation in the NPT ensemble^36^ of 12 ps in duration was run to relax the randomized ionic configuration at the target temperature of 1273 K using a time step of 0.25 fs. Following this, seven temperature-annealing steps were performed, each 40 ps in duration, from 1273 K up to 1626 K and then back down to 1273 K. This target temperature was selected to be consistent with the high melting point of some of the neat salts. Temperature and pressure were controlled using the Nosé–Hoover thermostat and barostat,^37,38^ each with a chain length of 5. Following simulated annealing, a 3 ns NPT simulation was run, with the last 2 ns used for production, from which radial distribution functions (g­(r)) and X-ray structure functions (S­(q)) were computed. We also computed the partial subcomponents of S­(q); details on the calculation of S­(q) and its partial subcomponents can be found in multiple prior publications.^19–22,31,32^ Both for equilibration and production, the time step, thermostat, and barostat time constants were set to 1.0, 500, and 2500 fs, respectively. For the annealing stages, the values were set to 1.0, 100, and 500 fs, respectively.

The same procedure was used for smaller boxes with 40 alkaline earth cations (Mg, Ca, Sr) and 80 chloride anions, and an extra KCl ion pair that, before starting our first-principles calculations, was replaced by Cl_2_ as was previously described in reference 14. Parameters for KCl were taken from Ishii et al.,^39^ cation–cation (K^+^-M^2+^) interaction parameters were taken to be the same as those for Mg^2+^-K^+^ as described in reference 29. For these smaller simulation boxes, the real-space cutoff was set to 12 Bohr (6.35 Å). A configuration matching the overall average simulation density was extracted from the last 1 ns of the production trajectory and used as input for additional ab initio molecular dynamics (AIMD) simulations.

### 2.2 First-Principles MD Simulations

Ab-initio molecular dynamics simulations were performed using the CP2K software (version 2023.2) in the NVT ensemble^40^ using the Nosé–Hoover thermostat^37^ and a 0.5 fs time step. All simulations were charge-neutral and spin-restricted. We used the Perdew–Burke–Ernzerhof (PBE) functional^41–43^ in conjunction with Grimme’s D3 dispersion corrections,^44^ the TZVP-MOLOPT basis sets,^45^ and GTH pseudopotentials^46^ as coded in CP2K. The planewave cutoff was set to 800 Ry, and the relative cutoff to 80 Ry.

For first-principles simulations of the MCl_2_ systems, the temperature was set to 1273 K. Input configurations for ab initio molecular dynamics (AIMD), derived from the PIM simulations, included 40 M^2+^ cations and 80 Cl^–^ counterions (M^2+^ = Mg^2+^, Ca^2+^, and Sr^2+^) and, for practical convenience, an additional KCl ion pair used as a placeholder and swapped for a Cl_2_ molecule before AIMD.^14^ For these MCl_2_ systems, the first 15 ps were taken as equilibration, and data was collected for production after that. Every 50 fs of the full 25 ps production run, molecular orbitals and electron densities were printed to be used in Bader charge analysis using software developed by the Henkelman group^47^ to compute atomic charges. To validate the Bader charge results, we also performed a Wannier analysis on selected snapshots, which confirmed the findings (data not shown).^48–50^

In the first-principles study, the case of BaCl_2_ was excluded because larger cationic species introduced significant DFT electron delocalization (instead of Cl_2_ or Cl_3_^–^ one could observe a large group of atoms with diminished valence charge); this was also observed to some extent for SrCl_2_, and to a lesser extent for CaCl_2_. The behavior is consistent with the known delocalization (self-interaction) error of semilocal GGA functionals under near-degenerate conditions, which can lead to transient, nonphysical charge smearing.^51^

Whereas the focus of this work is on the MCl_2_ sequence, we also wanted to make brief contact with the early literature in which including monovalent salts resulted in significant spectral shifts for Cl_2_ and in which the ${\mathrm{Cl}}_{2}+{\mathrm{Cl}}^{-}⇌{\mathrm{Cl}}_{3}^{-}$ equilibrium was proposed.^24^ For this purpose, using the same protocol already described in Section 2.1 we also separately equilibrated neat MgCl_2_ and a MgCl_2_–KCl mixture melt to a production temperature of 1173 K. Swapping out ions in the classically equilibrated system for a Cl_2_ molecule before running AIMD calculations resulted in first-principles periodic boxes with (1) 39 MgCl_2_ + 1 Cl_2_ and (2) 24 MgCl_2_ + 23 KCl + 1 Cl_2_; the first 20 ps in these first-principles runs were taken for equilibration. For these specific runs, a 1200 Ry cutoff was used, and the relative cutoff was set to 80 Ry; other simulation details were as described previously for the MCl_2_ systems. For all systems, simulation box lengths used for AIMD runs are provided in Table S1.

## 3 Results and Discussion

We begin this section by focusing on the structure of the MCl_2_ melts as seen from their computed structure functions, S­(q) shown in Figure 1. At first glance, the liquids look structurally very different. MgCl_2_ has a prepeak below 1 Å^–1^ that none of the other MCl_2_ systems have. BaCl_2_ and SrCl_2_ have a strong peak at around 1.5 Å^–1^ that neither CaCl_2_ nor MgCl_2_ have, and whereas peaks appear in sync for CaCl_2_, SrCl_2_, and BaCl_2_ above 2 Å^–1^, those for MgCl_2_ appear off-sync by about half a period! We will show that, except for the prepeak below 1 Å^–1^, which is a unique feature of MgCl_2_ that we have discussed in thorough detail in multiple prior publications,^27–29^ other apparent inconsistencies can be easily explained as issues of (1) X-ray contrast and (2) trends in cationic size.

**Figure 1:**
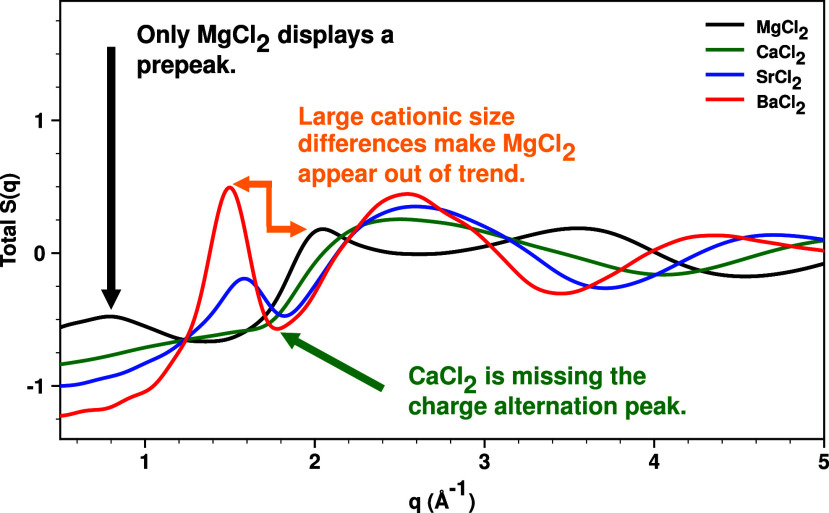
X-ray S­(q) functions derived from the large-box PIM simulations. At first glance, the functions may appear to be quite different, implying significant changes in melt structure. In reality, the changes are gradual, as can be derived from the partial subcomponents of S­(q) in Figure 2 and the trends in the metal-chloride subcomponents across melts in Figure 3.

We remind the reader that the hallmark feature of all molten salts and ionic liquids is a set of two peaks and one antipeak^19,31,52–54^ in the partial subcomponents of S­(q) that arise due to positive–negative charge alternation. The peaks appear at the typical reciprocal wavenumber associated with the distance between cations separated by anions or that of anions separated by cations, and the antipeak indicates that at such a distance from a cation, one is unlikely to find an anion and vice versa. These three features are highlighted with ovals in the panels of Figure 2.

**Figure 2:**
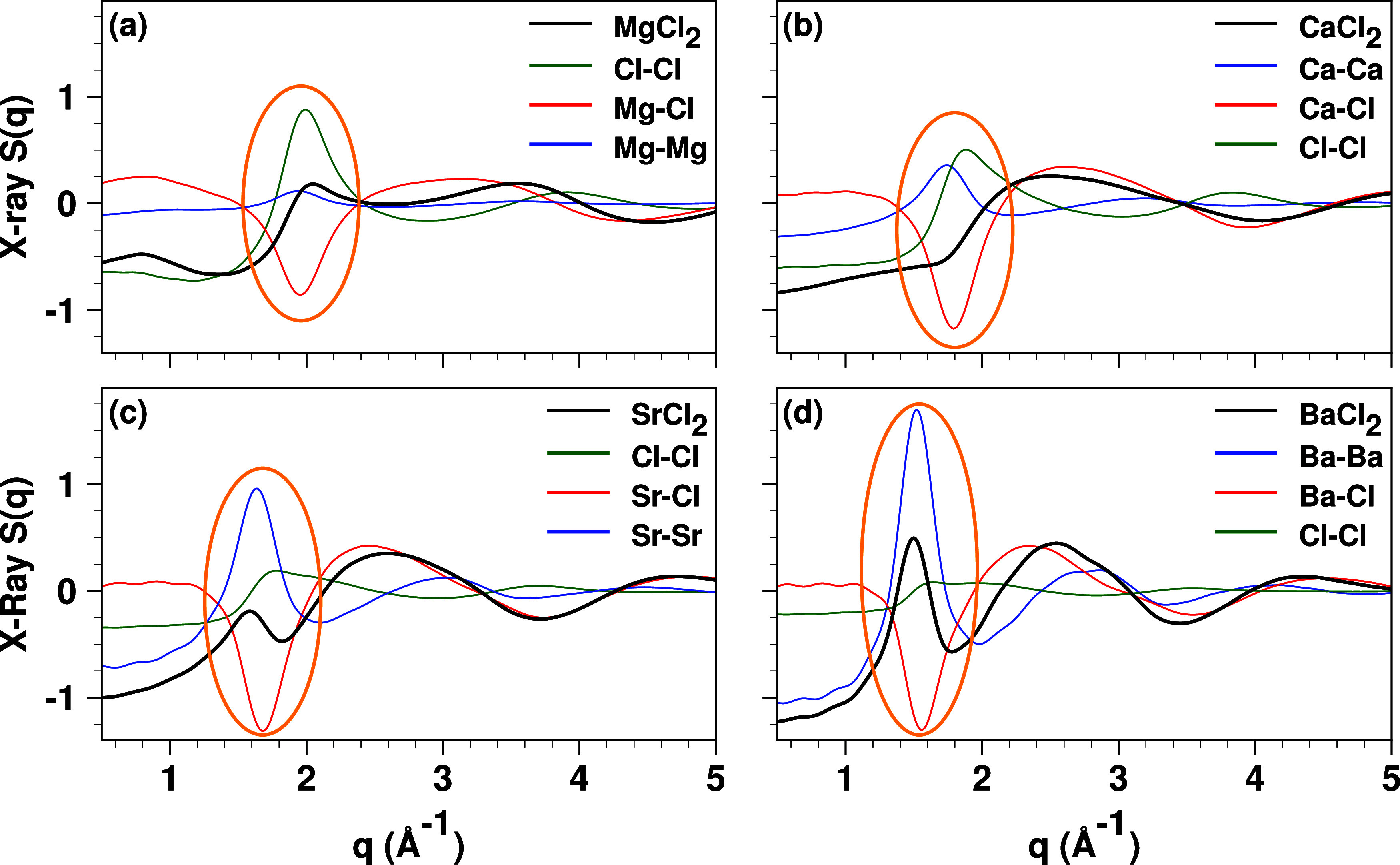
For MgCl_2_, CaCl_2_, SrCl_2_, and BaCl_2_ in panels a, b, c, and d, respectively, the total and partial subcomponents of S­(q). In each case, an oval highlights the charge alternation region, which occurs at different q values depending on the size of the metal ion.

Notice how, depending on the number of electrons in the cation and the anion, the metal-chloride antipeak (negative-going peak inside the oval) may just be of the right intensity to completely negate the two peaks associated with metal–metal and chloride–cloride charge interactions. We see this from the fact that the sum of the three subcomponents in each oval (the total S­(q)) shows a peak matching the oval region for MgCl_2_, SrCl_2_, and BaCl_2_, but not in the case of CaCl_2_. In other words, the fact that Figure 1 does not show a peak between 1.5 Å^–1^ and 2 Å^–1^ in the case of CaCl_2_ is completely fortuitous and due to poor X-ray contrast.

If we want to recover the expected structural trends associated with cationic size across the series (no missing features, no unrealistic shifts in periodicity), all that is needed is to focus on the metal-chloride subcomponent of S­(q) highlighted also with an oval in Figure 3.

**Figure 3:**
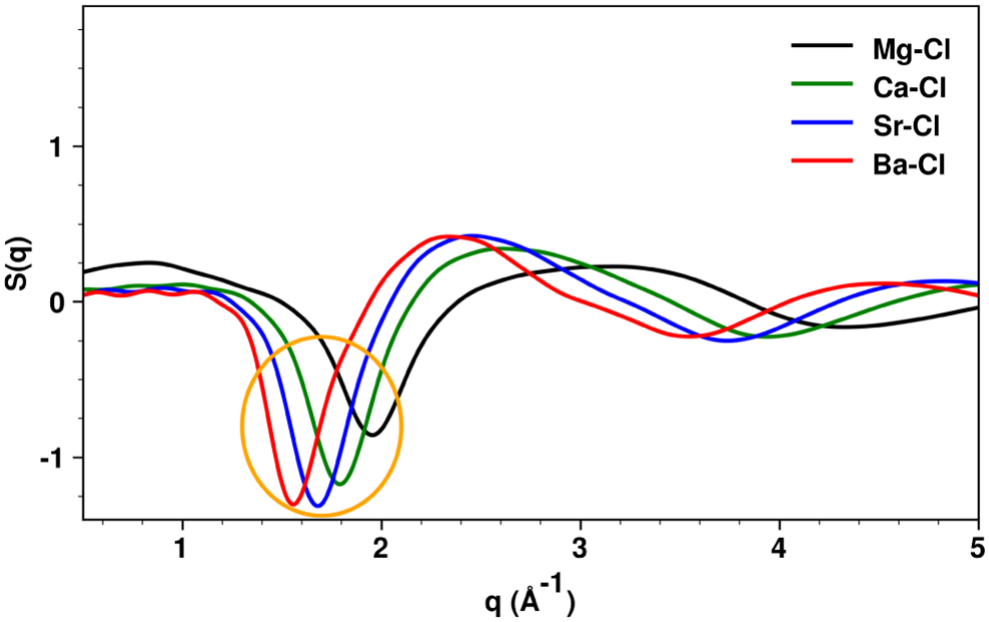
The metal-chloride partial subcomponent of S­(q) for each of the melts. The oval highlights the charge alternation antipeak region. Charge alternation antipeaks and other graph features follow smooth, expected trends with cationic size; compare this with the highly complex pattern of peaks in Figure 1.

Seen as a whole, we find time and again that peaks in the total X-ray S­(q) seldom tell a complete story, particularly for molten salts and ionic liquids where patterns of alternation cause massive cancellations. The cautionary tale is that simulations that can extract partial subcomponents of S­(q), or expensive experiments such as neutron scattering with isotopic substitution, are often needed to tease out a complete description of such systems.

We now turn our attention to the prepeak, the acidity of the cations, and the effect this has on the chlorobasicity of the anions. We have, in the past, explained that the preapeak in MgCl_2_ has to do with the distance between Cl^–^-decorated Mg^2+^ networks.^27–29^ More explicitly, the feature is related to the distance between Mg^2+^ ions that are relatively close but not close enough to share counterions. MgCl_2_ contains the most acidic cation in the MCl_2_ alkaline earth sequence and is the most structured melt, featuring a prepeak associated with intermediate-range order. A natural consequence of the acidity of the cation is that Cl^–^ in MgCl_2_ is the least chlorobasic. Put differently, Cl^–^ is committed to the Cl^–^-decorated Mg^2+^ networks, is significantly polarized (or shares significant electron density) with Mg^2+^ ions in the network, and is significantly bound to these.

Connecting these Lewis acid–base ideas with the findings the findings in reference 24, we initially analyze what happens when a Cl_2_ molecule is introduced into molten MgCl_2_ and contrast that with the case in which it is introduced into a MgCl_2_–KCl mixture, in our case with a concentration close to 50% KCl–MgCl_2_ (see Section 2). The bottom panels in Figure 4 show, as a function of time, what is essentially the number of valence electrons for chlorine-based species in each melt at 1173 K. In the case of the MgCl_2_ melt, we see that, for the most part, all species except for two have a value close to 8 on the *Y*-axis, corresponding to Cl^–^; the two species with a value close to 7 are Cl atoms forming the Cl_2_ molecule. The situation is radically different for the KCl–MgCl_2_ mixture, where (1) most of the time there are three Cl species with a diminished number of valence electrons, and (2) the actual atomic/ionic species forming ${\mathrm{Cl}}_{3}^{-}$ or Cl_2_ are in constant exchange. The top panels in the figure show that the distance (or bond length) between charge-diminished species is shorter when they are Cl_2_, and the vibrational pattern in the panels is also quite different when contrasting time regions dominated by Cl_2_ and ${\mathrm{Cl}}_{3}^{-}$ . Seen as a whole, the interpretation of spectroscopic observations in earlier experiments in terms of the ${\mathrm{Cl}}_{2}+{\mathrm{Cl}}^{-}⇌{\mathrm{Cl}}_{3}^{-}$ chemical equilibrium is supported by our calculations. In an environment with only acidic cations, the anions are less chlorobasic, and ${\mathrm{Cl}}_{3}^{-}$ seldom forms. The opposite is true in the presence of the weakly polarizing K^+^ cations.

**Figure 4:**
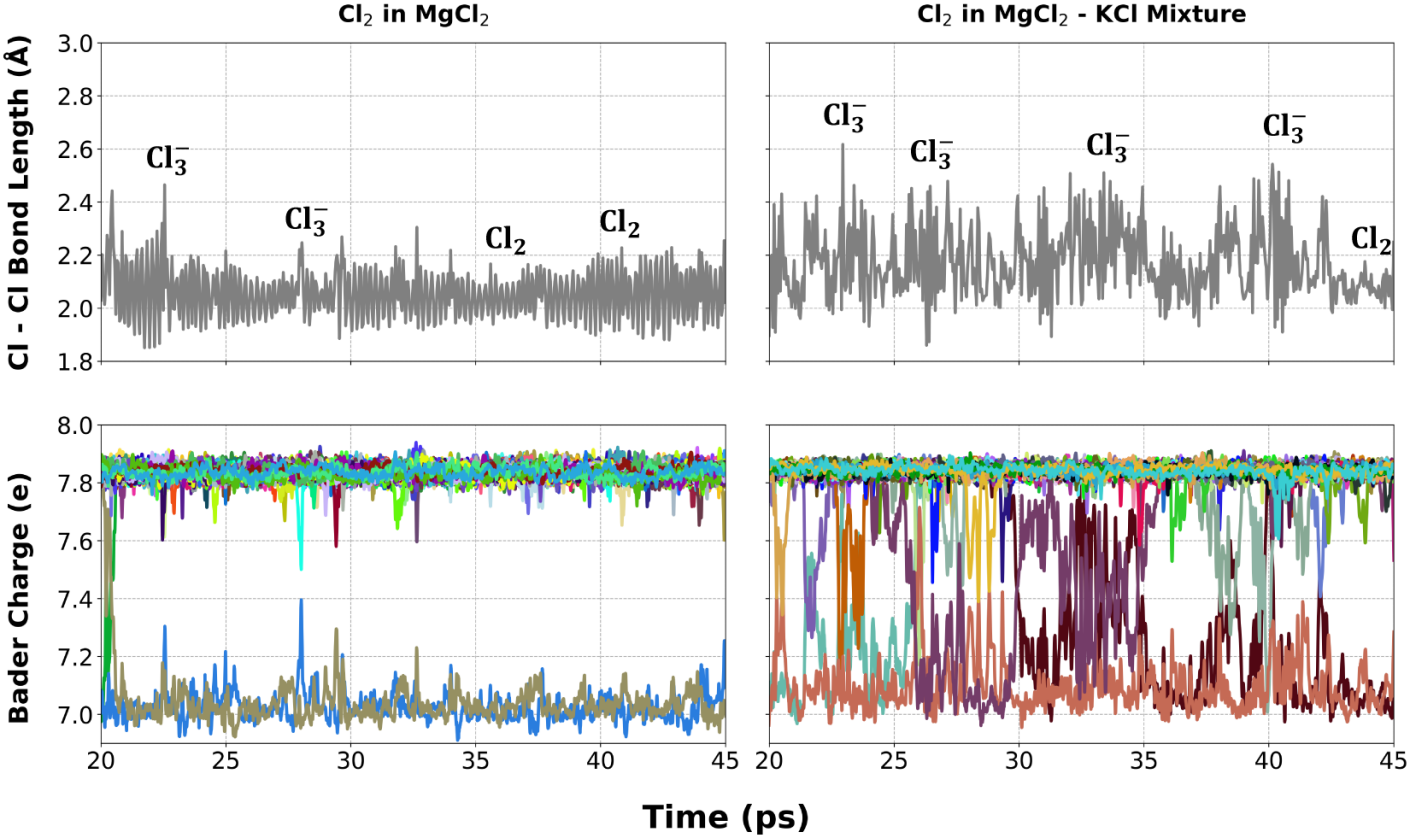
Typical time behavior of the ${\mathrm{Cl}}_{2}+{\mathrm{Cl}}^{-}⇌{\mathrm{Cl}}_{3}^{-}$ equilibrium process in neat MgCl_2_ and a ∼50% mixture of MgCl_2_ and KCl at 1173 K seen from (top) the distance*^a^* between the two Cl species with the lowest charge (nominally the two atoms in Cl_2_ or two atoms in ${\mathrm{Cl}}_{3}^{-}$ ) and (bottom) valence Bader charge analysis for all chlorine-based species, each with its own color. In the bottom plots a value close to 7 indicates Cl, whereas a value close to 8 indicates Cl^–^. In the top graphs, labels indicating Cl_2_ and ${\mathrm{Cl}}_{3}^{-}$ are to guide the eye; not every instance of Cl_2_ or ${\mathrm{Cl}}_{3}^{-}$ appears labeled. *^a^* *For context comparison, a gas-phase calculation (B3LYP-D3/def2svp) puts the* Cl_2_ *bond length at* ∼2.06 Å, *whereas symmetric* ${\mathrm{Cl}}_{3}^{-}$ *has bond lengths of* ∼2.38 Å *and an asymmetric version* *has bond lengths of* ∼2.28 Å *and* ∼3.00 Å, *respectively.*

Figure 4 already shows that in MgCl_2_ a radiation-produced Cl_2_ molecule should exist mostly as such. Exploring a higher temperature regime in Figure 5 we only see short periods in which ${\mathrm{Cl}}_{3}^{-}$ forms. The bottom left panel of Figure 5 shows a full sequence of the process ${\mathrm{Cl}}_{2}+{\mathrm{Cl}}^{-}\to {\mathrm{Cl}}_{3}^{-}\to {\mathrm{Cl}}_{2}+{\mathrm{Cl}}^{-}$ happening in the MgCl_2_ melt. Connections are shown between Cl-containing species and Mg^2+^ only when the distance is smaller than 2.7 Å. The distance is chosen because (1) it encompasses most of the first peak in g­(r) in the Mg^2+^–Cl^–^ case (see different g­(r) functions from the PIM model depicted in Figure S1), and (2) it s a short distance—but still within the first RDF peak—for Cl (in Cl_2_) and Mg^2+^, making the value relevant during reactive or chemical events (data not shown). In Figure 5, species close to a valence Bader charge value of 8 are Cl^–^ ions, whereas those close to 7 are chlorine atoms in Cl_2_ or ${\mathrm{Cl}}_{3}^{-}$ . When ${\mathrm{Cl}}_{3}^{-}$ forms, we see three species, denoted by their corresponding color code, that have valence significantly lower than 8. Commonly, one species remains close to a value of 7, and two others fluctuate with a typical value around 7.5, indicating charge sharing (this is akin to observations in the case of I_3_^–^ in other media^55,56^ ).

**Figure 5:**
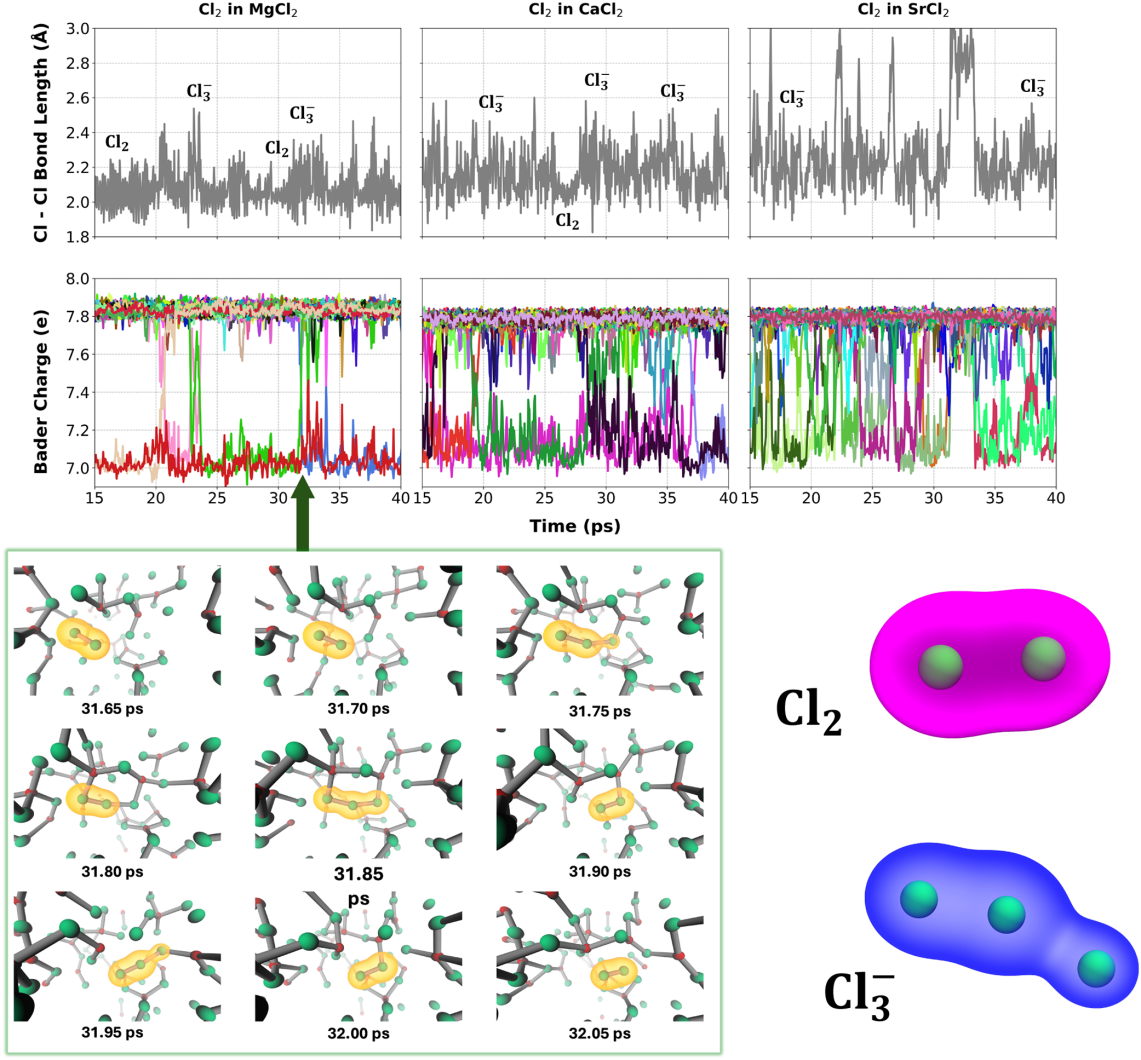
(Top) For systems labeled on the top and as a function of time, the distance between the two lowest charge Cl species (first row) and the valence number of electrons for Cl-containing species obtained from Bader analysis (second row). In the first row, labels indicating Cl_2_ and ${\mathrm{Cl}}_{3}^{-}$ are provided to guide the eye, and not every instance of Cl_2_ or ${\mathrm{Cl}}_{3}^{-}$ appears labeled. In the case of SrCl_2_, the plot includes not only exchange events involving ${\mathrm{Cl}}^{-}/{\mathrm{Cl}}_{2}/{\mathrm{Cl}}_{3}^{-}$ but also short periods of delocalization (see, for example, 31–33 ps and ref 51 for a discussion about DFT delocalization error). (Bottom) For the time region highlighted with an arrow, a ${\mathrm{Cl}}_{2}+{\mathrm{Cl}}^{-}\to {\mathrm{Cl}}_{3}^{-}\to {\mathrm{Cl}}_{2}+{\mathrm{Cl}}^{-}$ reaction sequence in the case of the MgCl_2_ melt (left panel) and typical σ­(s–s) bonding orbitals in the case of Cl_2_ and ${\mathrm{Cl}}_{3}^{-}$ obtained from the CaCl_2_ trajectory (right). In simulation snapshots, the figure depicts Cl-type species in green, Mg species in red, bonds drawn with a cutoff of 2.7 Å, and yellow orbitals are σ­(s–s) bonding. These orbitals help identify Cl_2_ and ${\mathrm{Cl}}_{3}^{-}$ species along the simulation because they are easy to track in the condensed-phase energy diagram; see ref 14.

The simulation snapshot sequence in Figure 5 shows that initially, Cl_2_ appears as a molecular species. At around 31.75 ps, when reactivity is about to take place, Cl_2_ recruits a Cl^–^ ion that is part of the MgCl_2_ network. We see this from the link between Cl^–^ and Mg^2+^ and from the σ­(s–s) bonding orbital that already starts to encompass some of the Mg^2+^-bound Cl^–^ ion. At later times, connections between ${\mathrm{Cl}}_{3}^{-}$ and the network form and break, but the ultimate result is a Cl_2_ molecular species that is detached from the network. Notice that in all melts, when the full ${\mathrm{Cl}}_{2}+{\mathrm{Cl}}^{-}\to {\mathrm{Cl}}_{3}^{-}\to {\mathrm{Cl}}_{2}+{\mathrm{Cl}}^{-}$ process occurs, the atoms making Cl_2_ at a later time may be different from those that made the molecule originally (we see this from color swaps in the Bader valence charge subfigures). In other words, if one were to bubble labeled Cl_2_ in any of these melts, the labeled Cl atoms would exchange with Cl^–^ ions in solution.

Just like in the case of the mixture between MgCl_2_ and KCl in Figure 4, Bader valence charge analysis, together with the distances plot in Figure 5 show that ${\mathrm{Cl}}_{3}^{-}$ is more likely to be observed in the case of CaCl_2_ when compared to ${\mathrm{MgCl}}_{2};{\mathrm{Cl}}_{3}^{-}$ is the dominant species in the case of SrCl_2_. Put differently, in MgCl_2_ the equilibrium ${\mathrm{Cl}}_{2}+{\mathrm{Cl}}^{-}⇌{\mathrm{Cl}}_{3}^{-}$ is dominated by the reactants, and by the product in the case of SrCl_2_; CaCl_2_ is an intermediate case between these two extremes.

## 4 Conclusions

Cl_2_, an expected product from radiation-induced electron detachment reactions in chloride-based molten salts (via nominal reactions Cl^–^ → e^–^ + Cl^·^, Cl^·^ + Cl^–^ → Cl_2_^·–^, 2Cl_2_^·–^ → Cl_2_ + 2Cl^–^),^8,10^ is in equilibrium with ${\mathrm{Cl}}_{3}^{-}$ (via ${\mathrm{Cl}}_{2}+{\mathrm{Cl}}^{-}⇌{\mathrm{Cl}}_{3}^{-}$ ) and this equilibrium shifts depending on the chlorobasicity of the melt. How free Cl^–^ is to donate electron density depends on the Lewis acidity of cations in the melt and how committed Cl^–^ is to their coordination. In MgCl_2_ melts, Cl_2_ remains for the most part a neutral molecular species. In neat chloride melts of the heavier alkaline earth cations (CaCl_2_, SrCl_2_), we notice instead a trend in which ${\mathrm{Cl}}_{3}^{-}$ is increasingly observed as we go down the series. The same type of effect can be seen when MgCl_2_ is mixed with the much less Lewis acidic K^+^ ion in KCl. Whether Cl_2_ or ${\mathrm{Cl}}_{3}^{-}$ is the most favored species in the melt is expected to be consequential to gas solubility (ions are expected to be more soluble in molten salts than neutral species) and the spatial distribution of radiation products (bulk vs interfaces). In terms of structure, the melts follow normal trends expected from cationic size, but one would not be able to infer that easily from X-ray scattering. S­(q) functions hardly follow any trend because of contrast issues, leaving the reader with an important cautionary tale.

## Supplementary Material



## Data Availability

Data sets for this article are made available within 30 days of the official acceptance date of this article by the journal in the Zenodo repository under the Digital Object Identifier (DOI): 10.5281/zenodo.20560046.

## Notes

Pair distribution functions for the different MCl_2_ systems from large PIM simulations and box sizes for first-principles simulations (PDF)

## References

[1] Forsberg C. W. (2006). Molten
Salt Reactors for a Sustainable Clean Energy Future. Prog. Nucl. Energy.

[2] Ding W., Shi H., Jianu A., Xiu Y., Bonk A., Weisenburger A., Bauer T. (2019). Molten Chloride Salts for Next Generation Concentrated Solar Power
Plants: Mitigation Strategies against Corrosion of Structural Materials. Sol. Energy Mater. Sol. Cells.

[3] Nguyen H. H., Huber K., Das D., Borah B., Emerson M. S., Knudtzon M., Wishart J. F., Blank D. A., Margulis C. J. (2025). Electrons
and Their Multiple Kinetic Fates in an Ionic Liquid. J. Am. Chem. Soc..

[4] Nguyen H. H., Bryantsev V. S., Margulis C. J. (2023). Are High-Temperature Molten Salts
Reactive with Excess Electrons? Case of ZnCl_2_. J. Phys. Chem. B.

[5] Margulis C. J., Annapureddy H. V. R., De Biase P. M., Coker D., Kohanoff J., Del Pópolo M.
G. (2011). Dry Excess Electrons
in Room-Temperature Ionic Liquids. J. Am. Chem.
Soc..

[6] Pikaev A. K., Makarov I. E., Zhukova T. N. (1982). Solvated
Electron in Irradiated Melts
of Alkaline Halides. Radiat. Phys. Chem..

[7] Akiyama R., Kitaichi M., Fujiwara T., Sawamura S. (1994). Short Lived Species
Produced in Pulse Irradiated Melts of LiF-KF and LiF-NaF-KF Eutectic
Mixtures. J. Nucl. Sci. Technol..

[8] Hagiwara H., Sawamura S., Sumiyoshi T., Katayama M. (1987). Pulse Radiolysis Study
of Transient Species in LiCl-KCl Melt. Int.
J. Radiat. Appl. Instrum. Part C.

[9] Sawamura S., Gȩbicki J. L., Mayer J., Kroh J. (1990). Pulse Radiolysis of
LiBr-KBr Melts. Optical Transient Absorption Spectra. Int. J. Radiat. Appl. Instrum. Part C.

[10] Iwamatsu K., Horne G. P., Gakhar R., Halstenberg P., Layne B., Pimblott S. M., Wishart J. F. (2022). Radiation-induced
Reaction Kinetics of Zn^2+^ with e*
_S_
*
^–^ and Cl_2_
^·–^in
Moten LiCl–KCl Eutectic at 400–600 ^◦^C. Phys. Chem. Chem. Phys..

[11] Iwamatsu K., Horne G. P., Ramos-Ballesteros A., Castro Baldivieso S., Conrad J. K., Woods M. E., Phillips W. C., LaVerne J. A., Pimblott S. M., Wishart J. F. (2026). Kinetics of Radiation-induced
Cr­(ii)
and Cr­(iii) Redox Chemistry in Molten LiCl–KCl Eutectic. Phys. Chem. Chem. Phys..

[12] Makarov I. E., Zhukova T. N., Pikaev A. K., Spitsyn V. I. (1982). Oxidizing Agents
Produced by Radiolysis of Alkali-Metal Halide Melts. Russ. Chem. Bull..

[13] Nguyen H. H., Huber K., Das D., Wishart J. F., Blank D. A., Margulis C. J. (2025). Ionic Liquids under Radiation and the Dimer Radical
Dicyanamide Anion. J. Phys. Chem. B.

[14] Nguyen H. H., Gibson L. D., Emerson M. S., Borah B., Roy S., Bryantsev V. S., Margulis C. J. (2025). Chlorine Gas and Anion Radical Reactivity
in Molten Salts and the Link to Chlorobasicity. Phys. Chem. Chem. Phys..

[15] Wu F., Xu C., Margulis C. J. (2018). Dynamics of an Excess Hole in the 1-methyl-1-butyl-pyrrolidinium
Dicyanamide Ionic-Liquid. J. Chem. Phys..

[16] Dhungana K. B., Wu F., Margulis C. J. (2017). Excess Electron and Hole in 1-Benzylpyridinium-Based
Ionic Liquids. J. Phys. Chem. B.

[17] Duffy J. A., Ingram M. D. (1971). Establishment of
an Optical Scale for Lewis Basicity
in Inorganic Oxyacids, Molten Salts, and Glasses. J. Am. Chem. Soc..

[18] Madden P. A., Wilson M. (1996). ‘Covalent’ Effects in ‘ionic’
systems. Chem. Soc. Rev..

[19] Emerson M. S., Ogbodo R., Margulis C. J. (2024). Spiers
Memorial Lecture: From Cold
to Hot, The Structure and Structural Dynamics of Dense Ionic Fluids. Faraday Discuss..

[20] Emerson M. S., Ivanov A. S., Gallington L. C., Maltsev D. S., Halstenberg P., Dai S., Roy S., Bryantsev V. S., Margulis C. J. (2024). Heterogeneous Structure,
Mechanisms of Counterion Exchange, and the Spacer Salt Effect in Complex
Molten Salt Mixtures Including LaCl_3_. J. Phys. Chem. B.

[21] Emerson M. S., Sharma S., Roy S., Bryantsev V. S., Ivanov A. S., Gakhar R., Woods M. E., Gallington L. C., Dai S., Maltsev D. S., Margulis C. J. (2022). Complete Description of the LaCl_3–_NaCl Melt Structure and the Concept of a Spacer Salt
That Causes Structural Heterogeneity. J. Am.
Chem. Soc..

[22] Roy S., Brehm M., Sharma S., Wu F., Maltsev D. S., Halstenberg P., Gallington L. C., Mahurin S. M., Dai S., Ivanov A. S., Margulis C. J., Bryantsev V. S. (2021). Unraveling
Local Structure of Molten Salts via X-ray Scattering, Raman Spectroscopy,
and Ab Initio Molecular Dynamics. J. Phys. Chem.
B.

[23] Roy S., Sharma S., Karunaratne W. V., Wu F., Gakhar R., Maltsev D. S., Halstenberg P., Abeykoon M., Gill S. K., Zhang Y., Mahurin S. M., Dai S., Bryantsev V. S., Margulis C. J., Ivanov A. S. (2021). X-ray Scattering Reveals Ion Clustering
of Dilute Chromium Species in Molten Chloride Medium. Chem. Sci..

[24] Andresen, R. E. ; Østvold, T. ; Øye, H. A. Solubility of Chlorine in Molten Chlorides. Proceedings of the 1st International Symposium on Molten Salts; IOPscience, 1976, 1976-6, 1−111. 10.1149/197606.0111PV

[25] Biggin S., Enderby J. E. (1981). The structure of molten zinc chloride. J. Phys. C: Solid State Phys..

[26] Salmon P. S. (1992). The structure
of molten and glassy 2:1 binary systems: an approach using the Bhatia—Thornton
formalism. Proc. R. Soc. London, Ser. A.

[27] Sharma S., Emerson M. S., Wu F., Wang H., Maginn E. J., Margulis C. J. (2020). SEM-Drude Model
for the Accurate and Efficient Simulation
of MgCl_2_–KCl Mixtures in the Condensed Phase. J. Phys. Chem. A.

[28] Wu F., Sharma S., Roy S., Halstenberg P., Gallington L. C., Mahurin S. M., Dai S., Bryantsev V. S., Ivanov A. S., Margulis C. J. (2020). Temperature Dependence of Short and
Intermediate Range Order in Molten MgCl_2_ and Its Mixture
with KCl. J. Phys. Chem. B.

[29] Wu F., Roy S., Ivanov A. S., Gill S. K., Topsakal M., Dooryhee E., Abeykoon M., Kwon G., Gallington L. C., Halstenberg P., Layne B., Ishii Y., Mahurin S. M., Dai S., Bryantsev V. S., Margulis C. J. (2019). Elucidating Ionic Correlations Beyond
Simple Charge Alternation in Molten MgCl_2_–KCl Mixtures. J. Phys. Chem. Lett..

[30] Wang H., DeFever R. S., Zhang Y., Wu F., Roy S., Bryantsev V. S., Margulis C. J., Maginn E. J. (2020). Comparison of Fixed
Charge and Polarizable Models for Predicting the Structural, Thermodynamic,
and Transport Properties of Molten Alkali Chlorides. J. Chem. Phys..

[31] Roy S., Wu F., Wang H., Ivanov A. S., Sharma S., Halstenberg P., Gill S. K., Milinda Abeykoon A. M., Kwon G., Topsakal M., Layne B., Sasaki K., Zhang Y., Mahurin S. M., Dai S., Margulis C. J., Maginn E. J., Bryantsev V. S. (2020). Structure
and Dynamics of the Molten Alkali-Chloride Salts from an X-ray, Simulation,
and Rate Theory Perspective. Phys. Chem. Chem.
Phys..

[32] Sharma S., Ivanov A. S., Margulis C. J. (2021). A Brief
Guide to the Structure of
High-Temperature Molten Salts and Key Aspects Making Them Different
from Their Low-Temperature Relatives, the Ionic Liquids. J. Phys. Chem. B.

[33] Ishii Y., Kiko S., Ohtori N. (2024). Analysis of the Transport
Properties
of Alkaline-Earth Halides MX_2_ (M = Ca, Sr, Ba, and X =
F, Cl, Br) by Simulation with a Polarizable Ion Model. Electrochemistry.

[34] Marin-Lafléche A., Haefele M., Scalfi L., Coretti A., Dufils T., Jeanmairet G., Reed S., Serva A., Berthin R., Bacon C., Bonella S., Rotenberg B., Madden P., Salanne M. (2020). MetalWalls: A Classical Molecular
Dynamics Software Dedicated to the Simulation of Electrochemical Systems. J. Open Source Softw..

[35] Krüger D. M., Kamerlin S. C. L. (2017). Micelle Maker: An Online Tool for Generating Equilibrated
Micelles as Direct Input for Molecular Dynamics Simulations. ACS Omega.

[36] Martyna G. J., Tobias D. J., Klein M. L. (1994). Constant Pressure
Molecular Dynamics
Algorithms. J. Chem. Phys..

[37] Nosé S. (1984). A Unified
Formulation of the Constant Temperature Molecular Dynamics Methods. J. Chem. Phys..

[38] Hoover W. G. (1985). Canonical
Dynamics: Equilibrium Phase-Space Distributions. Phys. Rev. A.

[39] Ishii Y., Kasai S., Salanne M., Ohtori N. (2015). Transport Coefficients
and the Stokes–Einstein Relation in Molten Alkali Halides with
Polarisable Ion Model. Mol. Phys..

[40] Kühne T. D., Iannuzzi M., Del Ben M., Rybkin V. V., Seewald P., Stein F., Laino T., Khaliullin R. Z., Schütt O., Schiffmann F. (2020). CP2K: An Electronic
Structure and Molecular Dynamics Software Package - Quickstep: Efficient
and Accurate Electronic Structure Calculations. J. Chem. Phys..

[41] Perdew J. P., Burke K., Ernzerhof M. (1996). Generalized
Gradient Approximation
Made Simple. Phys. Rev. Lett..

[42] Zhang Y., Yang W. (1998). Comment on “Generalized
Gradient Approximation Made Simple”. Phys. Rev. Lett..

[43] Perdew J. P., Burke K., Ernzerhof M. (1998). Perdew, burke,
and ernzerhof reply. Phys. Rev. Lett..

[44] Grimme S., Antony J., Ehrlich S., Krieg H. (2010). A Consistent and Accurate
Ab Initio Parametrization of Density Functional Dispersion Correction
(DFT-D) for the 94 Elements H-Pu. J. Chem. Phys..

[45] Vandevondele J., Hutter J. (2007). Gaussian Basis Sets for Accurate Calculations on Molecular
Systems in Gas and Condensed Phases. J. Chem.
Phys..

[46] Goedecker S., Teter M., Hutter J. (1996). Separable
Dual-Space Gaussian Pseudopotentials. Phys.
Rev. B.

[47] Sanville E., Kenny S. D., Smith R., Henkelman G. (2007). Improved Grid–Based
Algorithm for Bader Charge Allocation. J. Comput.
Chem..

[48] Koch D., Manzhos S. (2017). On the Charge State
of Titanium in Titanium Dioxide. J. Phys. Chem.
Lett..

[49] Walsh A., Sokol A. A., Buckeridge J., Scanlon D. O., Catlow C. R. A. (2017). Electron
Counting in Solids: Oxidation States, Partial Charges, and Ionicity. J. Phys. Chem. Lett..

[50] Jiang L., Levchenko S. V., Rappe A. M. (2012). Rigorous Definition
of Oxidation
States of Ions in Solids. Phys. Rev. Lett..

[51] Bryenton K. R., Adeleke A. A., Dale S. G., Johnson E. R. (2023). Delocalization error:
The greatest outstanding challenge in density-functional theory. WIREs Comput. Mol. Sci..

[52] Kashyap H. K., Hettige J. J., Annapureddy H. V. R., Margulis C. J. (2012). SAXS Anti-Peaks
Reveal the Length-Scales of Dual Positive–Negative and Polar–Apolar
Ordering in Room-Temperature Ionic Liquids. Chem. Commun..

[53] Wu F., Karunaratne W. V., Margulis C. J. (2019). Ionic Liquid Mixture at the Vacuum
Interface and the Peaks and Antipeaks Analysis of X-ray Reflectivity. J. Phys. Chem. C.

[54] Araque J. C., Hettige J. J., Margulis C. J. (2015). Modern Room Temperature
Ionic Liquids,
a Simple Guide to Understanding Their Structure and How It May Relate
to Dynamics. J. Phys. Chem. B.

[55] Margulis C. J., Coker D. F., Lynden-Bell R. M. (2001). Monte Carlo
Study of Symmetry Breaking
of I_3_
^–^ in Aqueous Solution using a Multistate
Diabatic Hamiltonian. J. Chem. Phys..

[56] Margulis C. J., Coker D. F., Lynden-Bell R. M. (2001). Symmetry
Breaking of the Triiodide
Ion in Acetonitrile Solution. Chem. Phys. Lett..

