# Supplementary material for: Chlorine Gas as a Lewis Acid–Base Probe for Molten Salts of Divalent Metal Ions
Source: J Phys Chem B. 2026 Jun 12;130(25):6376–82. doi: 10.1021/acs.jpcb.6c01313 (PMC13312442; doi:10.1021/acs.jpcb.6c01313)
Supplement: Supplementary file 1 [file jp6c01313_si_001.pdf]

Supporting Information: Chlorine Gas as a Lewis Acid-Base Probe for Molten Salts of Divalent Metal Ions

Yang Chen,† Matthew S. Emerson,‡ Hung H. Nguyen,† Raphael Ogbodo,† Vyacheslav S. Bryantsev,*,¶ James F. Wishart,*,‡ and Claudio J. Margulis*,†

†*Department of Chemistry, The University of Iowa, Iowa City, IA 52242, United States*

‡*Chemistry Department, Brookhaven National Laboratory, NY 55455, United States*

¶*Chemical Sciences Division, Oak Ridge National Laboratory, Oak Ridge, TN 37831, United States*

E-mail: bryantsevv@ornl.gov; wishart@bnl.gov; claudio-margulis@uiowa.edu

Contents

1 Supporting Tables and Figures S2

1 Supporting Tables and Figures

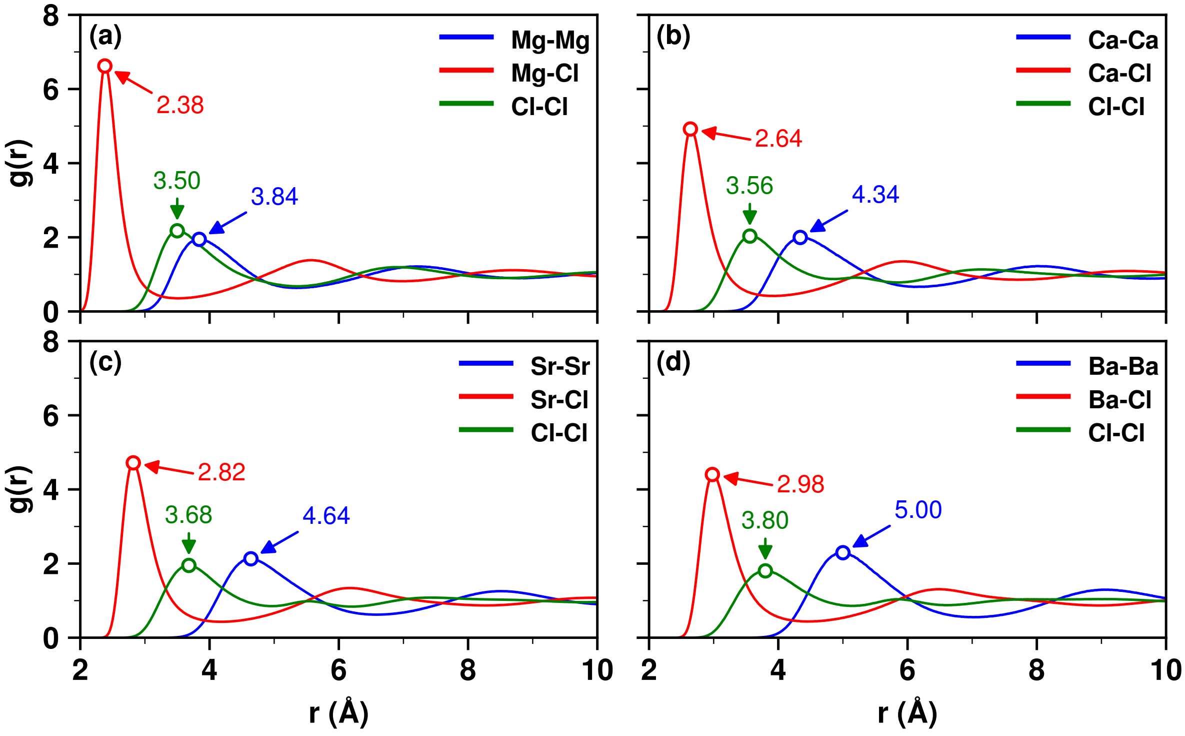

Figure S1: Pair distribution functions from PIM simulations.

Table S1: Box lengths for first principles MD runs at 1273 K unless explicitly indicated.

System	Cubic box length (Å)
$MgCl_2$	16.0105
$CaCl_2$	15.5914
$SrCl_2$	16.0033
$BaCl_2$	16.5230
$MgCl_2$ (1173K)	15.8595
$MgCl_2$-KCl (1173K)	16.3019
